# Advanced interventions in the pre-hospital resuscitation of patients with non-compressible haemorrhage after penetrating injuries

**DOI:** 10.1186/s13054-022-04052-7

**Published:** 2022-06-20

**Authors:** E. ter Avest, L. Carenzo, R. A. Lendrum, M. D. Christian, R. M. Lyon, C. Coniglio, M. Rehn, D. J. Lockey, Z. B. Perkins

**Affiliations:** 1 London’s Air Ambulance and Bart’s Health NHS Trust, Royal London Hospital, 17th floor, London, E1 1FR UK; 2 Air Ambulance Kent Surrey and Sussex, Hanger 10 Redhill Aerodrome, Redhill, UK; 3 Department of Emergency Medicine, University Medical Centre Groningen, University of Groningen, Groningen, The Netherlands; 4 Department of Anesthesia and Intensive Care Medicine, IRCCS Humanitas Research Hospital, Rozzano, Milan, Italy; 5 Department of Perioperative Medicine, St Bartholomew’s Hospital, London, UK; 6 BC Emergency Health Services, Provincial Health Services Authority, Vancouver, BC Canada; 7 School of Health Sciences, University of Surrey, Surrey, UK; 8 Department of Anesthesia, Intensive Care and Pre-Hospital Emergency Medical Services, Maggiore Hospital Carlo Alberto Pizzardi, Bologna, Italy; 9 The Norwegian Air Ambulance Foundation, Oslo, Norway; 10 Air Ambulance Department, Oslo University Hospital, Oslo, Norway; 11 Faculty of Health Sciences, University of Stavanger, Stavanger, Norway; 12 Queen Mary University, London, UK; 13 Centre for Trauma Sciences, Queen Mary University of London, London, UK; 14 Present Address: Division of Critical Care Medicine, Department of Medicine, University of British Columbia, Vancouver, BC Canada

**Keywords:** Penetrating injuries, Pre-hospital, Helicopter emergency medical services (HEMS), Interventions

## Abstract

**Abstract:**

Early haemorrhage control and minimizing the time to definitive care have long been the cornerstones of therapy for patients exsanguinating from non-compressible haemorrhage (NCH) after penetrating injuries, as only basic treatment could be provided on scene. However, more recently, advanced on-scene treatments such as the transfusion of blood products, resuscitative thoracotomy (RT) and resuscitative endovascular balloon occlusion of the aorta (REBOA) have become available in a small number of pre-hospital critical care teams. Although these advanced techniques are included in the current traumatic cardiac arrest algorithm of the European Resuscitation Council (ERC), published in 2021, clear guidance on the practical application of these techniques in the pre-hospital setting is scarce. This paper provides a scoping review on how these advanced techniques can be incorporated into practice for the resuscitation of patients exsanguinating from NCH after penetrating injuries, based on available literature and the collective experience of several helicopter emergency medical services (HEMS) across Europe who have introduced these advanced resuscitation interventions into routine practice.

**Graphical Abstract:**

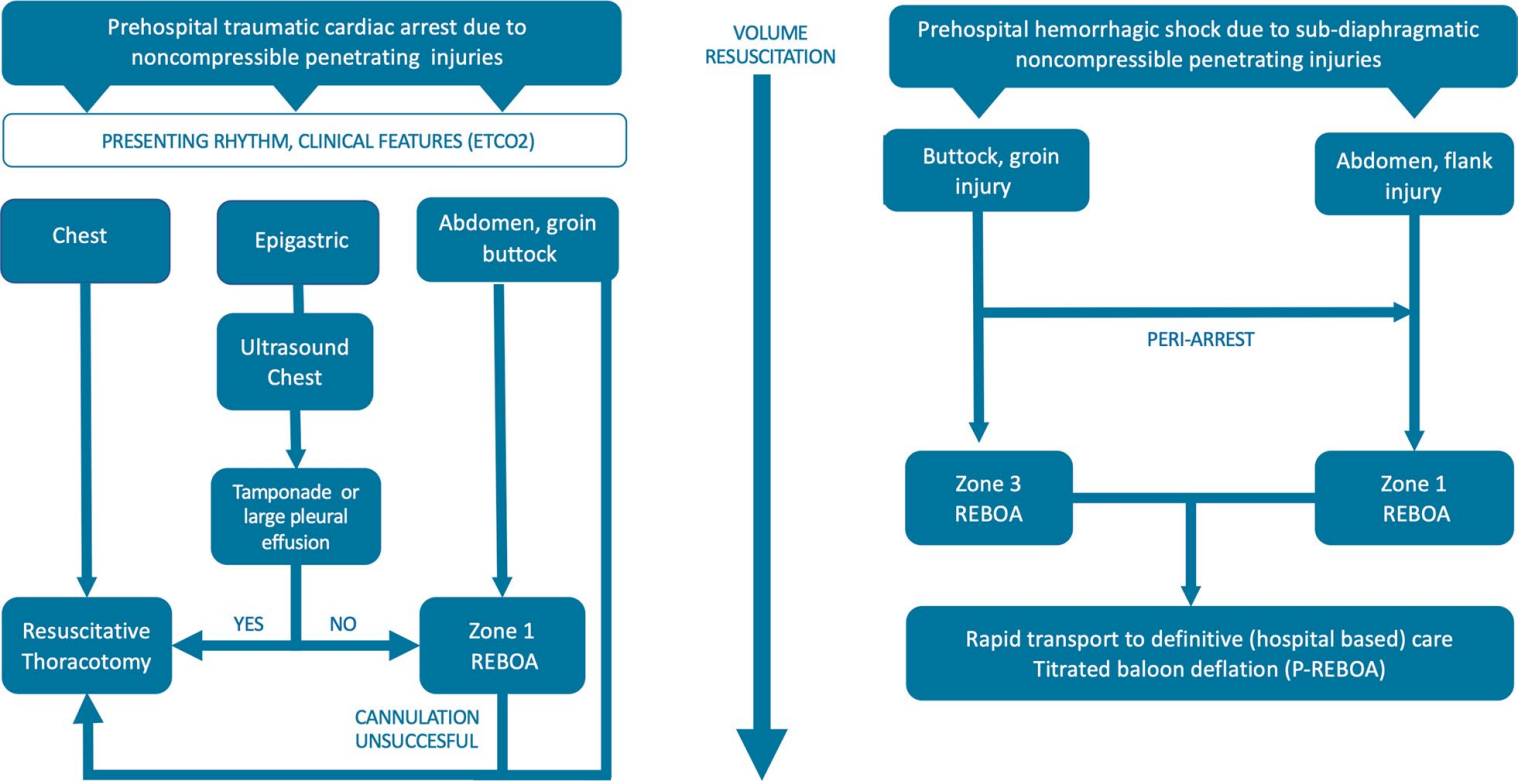

## Introduction

Trauma is a major cause of death and disability worldwide in all age groups [1, 2]. Penetrating trauma represents approximately 5–12% of the overall trauma case load [3–8]. In most countries, sharp object penetrating injuries (mainly knife stab wounds) constitute the majority of penetrating trauma [7, 8], although a rising incidence of ballistic injuries (gunshot wounds (GSW’s) has been reported in some countries [9]. Minimizing the time to definitive in-hospital, surgical care has long been the cornerstone of pre-hospital therapy for all patients with penetrating injuries, especially when non-compressible haemorrhage (NCH) was suspected. Historically, these patients were transported to hospital as quickly as possible, as minimal treatment could be provided on scene.

While minimizing the time to definitive care remains a priority, advanced pre-hospital interventions such as blood product administration [10, 11], resuscitative thoracotomy (RT) [12] and resuscitative endovascular balloon occlusion of the aorta (REBOA) [13] are now available in some pre-hospital critical care teams to treat patients with penetrating injuries. These techniques offer the potential to initiate earlier advanced trauma resuscitation in the pre-hospital phase of care and thereby have the potential to prevent death from pre-hospital exsanguination [14].

The revised traumatic cardiac arrest algorithm of the European Resuscitation Council (ERC) published in 2021 [15] mentions that interventions such as REBOA and RT can be considered to address potentially reversible causes of a traumatic cardiac arrest (in parallel to other interventions). However, specific guidance on when to deploy these techniques in the pre-hospital setting is currently lacking, leaving pre-hospital clinicians with complex decisions under significant time pressure and high levels of uncertainty: available information is often limited, anatomical injuries are often unknown, and various (non) technical and logistical challenges must be overcome. Furthermore, deployment of these techniques is not without risk, as potential complications are numerous and can be life threatening [16, 17].

This paper aims to provide a scoping review on when advanced techniques such as blood product transfusion, RT and REBOA can be utilized in the pre-hospital setting for the resuscitation of patients with NCH due to non-ballistic penetrating injuries, based on available literature and the collective experience of several helicopter emergency medical services (HEMS) across Europe who have introduced these advanced resuscitation interventions into routine practice.

## Advanced pre-hospital treatment interventions

The primary treatment goal for all patients with NCH in the pre-hospital setting is to maintain or restore adequate coronary (and therefore cerebral) perfusion until definitive (in-hospital) haemorrhage control can be achieved. Blood product transfusion, RT and REBOA can be used to facilitate cardiac resuscitation to achieve this goal in patients who are otherwise unlikely to survive the pre-hospital phase of care.

While blood transfusion may restore effective circulating volume, aortic occlusion by RT or REBOAs’ mechanism of action is to augment proximal diastolic blood pressure (and therefore coronary perfusion) [18] and resuscitate myocardial ischaemia, the final common pathway to cardiac arrest from exsanguination. It may also limit the extent of ischaemic cardiac injury which has been observed to kill patients even after haemorrhage control has been achieved [19]. In addition, both techniques reduce pressures below the level of the aortic occlusion, thereby limiting blood loss [20, 21]. RT further offers the opportunity to treat cardiac tamponade and control intrathoracic haemorrhage. It is beyond the scope of this article to describe *how* both techniques should be applied, as this has been described in detail previously [13, 22]. In the remainder of this article, we will discuss *when* each of these techniques should be considered in patients with a traumatic cardiac arrest (TCA; first section) or patients with a profound haemorragic shock (second section) as a result of NCH from non-ballistic penetrating injuries.

## Patients in traumatic cardiac arrest

**Fig. 1:**
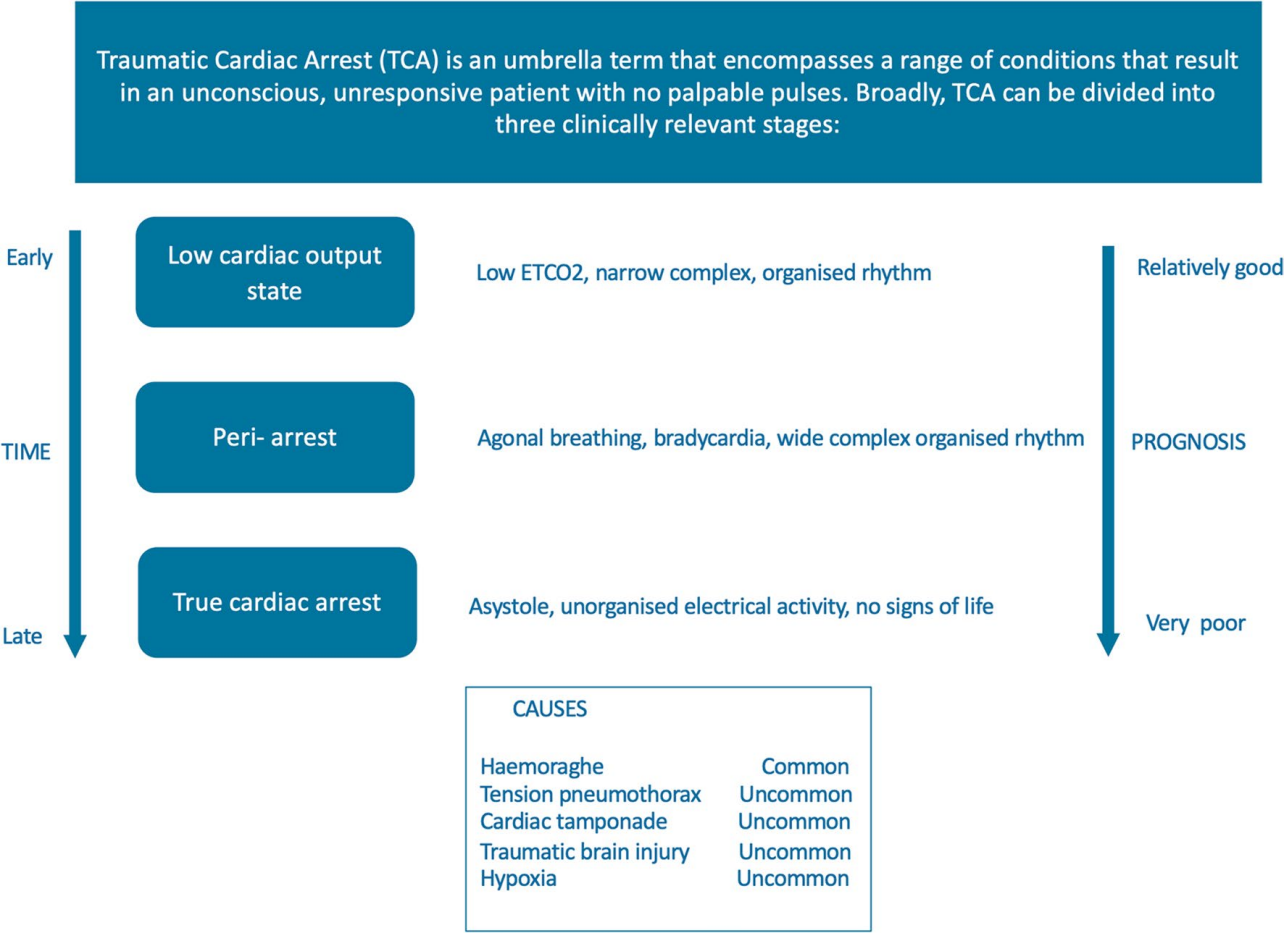
Traumatic cardiac arrest (TCA)

**Fig. 2:**
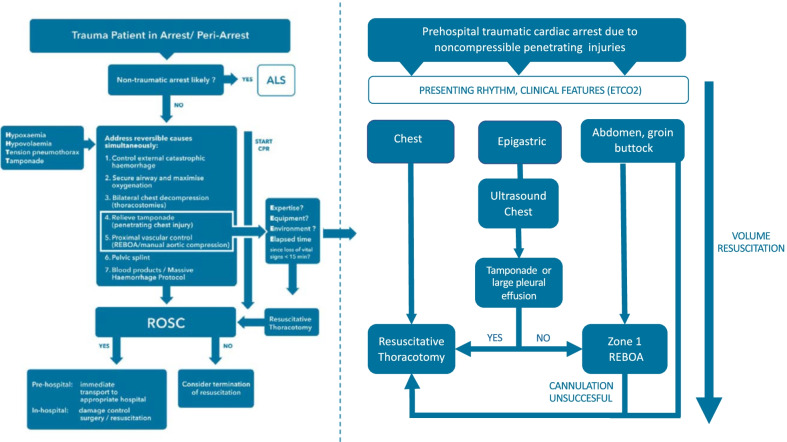
Options for cardiac resuscitation of patients in traumatic cardiac arrest (TCA) due to non-compressible haemorrhage from non-ballistic penetrating injuries: Addition to the 2021 ERC guidelines [15] on the treatment of patients in traumatic cardiac arrest (original figure adapted with permission)

When patients present in TCA (Fig. 1) after non-ballistic penetrating injuries, it is important to rapidly establish the time of arrest to inform subsequent decision-making. It can be extremely challenging to establish an accurate timeline after arrival on scene, and a concise handover from the emergency medical service (EMS) personnel on scene is invaluable. In general, patients with a prolonged period of cardiac arrest are unlikely to benefit from advanced invasive pre-hospital interventions such as a RT [12]. The ERC guideline and other guidelines suggest to consider RT when other reversible causes have been addressed and a maximum of 10–15 min has been elapsed since vital signs were lost (Fig. 2) [15, 23, 24]. However, the evidence supporting these cut-offs is weak and timings are only general guidance: patients who have exsanguinated and are in cardiac arrest with asystole or a wide complex disorganized rhythm have an extremely poor prognosis, whereas patients who have a low flow state with an organized narrow complex rhythm and/or a (relatively) high ETCO2 may have the potential for a good outcome and warrant ongoing aggressive resuscitation attempts [25–28]. As evidence-based cut-off values for ETCO2 are lacking to inform decision-making, a trend in ETCO2 values is more important than absolute values to inform decision-making.

At all times, decision-making must continue in an efficient manner: time should not be wasted establishing monitoring for this purpose if it is not already in place. If there is any doubt about timings, a rapid point of care ultrasonography (POCUS) (to confirm if organized cardiac activity is present) can contribute to decision-making when there is reasonable doubt about the time of arrest in the absence of an organized rhythm, but no time should be wasted doing this when an organized rhythm is present [29].

### Haemorrhage control and cardiac resuscitation

Current ERC guidelines [15] suggest that both RT and REBOA can be considered to facilitate cardiac resuscitation in patients with a TCA from NCH. The decision whether to proceed with RT or REBOA is dependent on available resources and training, but also on the mechanism of the injury and anatomical location of the injuries (Fig. 1).

#### Chest injuries

When patients suffer from isolated penetrating injury to the chest resulting in a TCA, RT offers the opportunity to relieve a cardiac tamponade and to obtain haemorrhage control by direct treatment of intrathoracic injuries. For this purpose, the clamshell technique maximizes exposure to facilitate identification and treatment of injuries [22, 30, 31]. Two-handed internal cardiac massage in combination with aortic cross-clamping and aggressive volume replacement increases pre-load, stroke volume (SV) and afterload, contributing to improved myocardial perfusion and oxygen delivery [18]. RT is most effective for patients with cardiac tamponade following penetrating trauma and a short period of TCA [12]. In these patients, swift decision-making is warranted and a low threshold to perform a RT could be justified. Although the overall mortality of patients with a pre-hospital RT is high as a prolonged no-flow time, aortic cross-clamping and hypothermia often result in irreversible trauma-induced coagulopathy and/or multi-organ failure [32, 33], the survival in patients who have a cardiac tamponade and a short TCA duration approaches 20% [12].

#### Abdominal, groin or buttock injuries

When penetrating injuries are located below the diaphragm and the patient is in TCA, both RT and REBOA can be considered. If RT is performed, it is with the aim of occluding the descending aorta by manual compression (or by cross-clamping) as a pre-laparotomy rescue manoeuvre [29]. Alternatively, REBOA can be used in combination with volume resuscitation, external cardiac massage and low-dose titrated vasopressors [13]. As with aortic cross clamping, in addition to reducing distal pressures and therefore blood loss [20, 31] an important effect of REBOA is augmentation of the diastolic and mean arterial blood pressure proximal to the balloon, thereby improving coronary and cerebral perfusion. In TCA, this effect is likely best achieved with zone I REBOA (with supra-coeliac balloon inflation) rather than zone III REBOA (with a balloon position below the renal arteries) [34, 35]. The increase in coronary perfusion may prevent cardiac arrest due to profound cardiac ischemia in deeply hypovolemic patients. It should be noted though that in patients with less profound shock, this beneficial effect should be balanced against the risk of creating a higher afterload and increased myocardial oxygen consumption.

One of the key challenges of REBOA in patients in TCA is the cannulation of the common femoral artery. Although previous studies have shown the feasibility of intra-arrest cannulation in patients who had suffered a medical arrest [36–38], cannulation may be more challenging in significantly hypovolemic patients, even when using ultrasound. Surgical cut-down to expose the femoral vessels may be considered by proficient providers to facilitate cannulation in this instance [39]. However, this procedure is not part of the skill set of most pre-hospital providers and comes with the risk of significant complications. Irrespective of the approach, it is important to limit the time allowed for cannulation and to consider conversion to RT if cannulation is not successful. Although REBOA seems to be a promising new technique for patients in TCA due to subdiaphragmatic penetrating injuries, it should be noted that pre-hospital outcome data on REBOA for this indication are not yet available.

#### Epigastric injuries

Epigastric injuries are particularly challenging to pre-hospital care providers, as they are at the junction of the abdominal and thoracic cavity. In these instances, rapid POCUS in addition to clinical assessment can help the pre-hospital clinician to decide whether to proceed with RT or with REBOA [28]. If a tamponade or a large pleural effusion is detected, RT is considered, whereas in the absence of these, an abdominal injury is more likely the cause of the hypovolemic arrest, and REBOA may be used to resuscitate the heart and to gain haemorrhage control as described above [40, 41].

#### Mixed injuries

When there are multiple penetrating injuries of both chest and abdomen/groin, an informed decision needs to be made about the most likely source of bleeding causing the TCA [40, 41]. This can be extremely challenging, and information about the mechanisms of injury, the suspected trajectory of injury and POCUS findings may help to establish the most likely primary source of bleeding and which of RT or REBOA is indicated. When concomitant penetrating head injuries are present, RT or REBOA are likely futile, and one should have a high threshold to perform any of these procedures.

### The role of external cardiac massage and vasoactive drugs

External cardiac massage is often of limited benefit where cardiac arrest is due to hypovolemia or a tamponade, as it relies on the passive filling of the heart during the recoil phase after each compression [42]. During traumatic causes of cardiac arrest, such as exsanguination, tamponade and tension pneumothorax, preload and diastolic ventricular filling are significantly reduced, and hence the ejection fraction during CPR (around 30% in euvolemic individuals) [43] is likely to be very low. The exemption to this rule is patients in cardiac arrest from sub-diaphragmatic exsanguination in whom REBOA is performed. Following balloon inflation, the reduction in blood loss may allow ventricular filling and when combined with external cardiac massage lead to an increase in coronary perfusion pressure and myocardial oxygen delivery.

Standard resuscitation doses of adrenaline at fixed intervals should be avoided as no survival benefit has been demonstrated in patients with a hypovolemic cardiac arrest [44]. If external compression CPR is provided, this should ideally be done with an active compression-decompression device that can generate negative pressure in the upstroke, thereby increasing preload.

### Post-ROSC care

To maximize chances of ROSC optimal quality CPR, especially cardiac massage, should be provided. Transporting patients in-arrest to hospital will compromise this quality and is therefore not preferable when the advanced treatments as described have already been delivered on scene. When ROSC is obtained, rapid transfer to definitive (hospital based) care is warranted for all patients. Distal perfusion should be restored as soon as possible after a period of heart and volume resuscitation, as prolonged complete aortic occlusion is unsurvivable due to the distal ischaemia created [45]. The actual period of occlusion that is survivable for zone I and III REBOA is currently unknown. When a REBOA balloon is deployed, titrated balloon deflation, a technique termed partial REBOA (P-REBOA) can be introduced [46]..P-REBOA is commenced 10–20 min after balloon inflation if the patient’s haemodynamics make it feasible, after targeted volume resuscitation. The aim is to transition to partial occlusion by allowing some blood flow distal to the site of balloon occlusion, to mitigate the ischaemia created, using distal arterial pressures as a surrogate for flow ^47^.

## Patients presenting with profound shock

Although a diagnosis of hypovolemic shock due to blood loss should be assumed in all patients presenting in shock with penetrating injuries, other causes of shock, such as tension pneumothorax or a pericardial tamponade, may also exist, especially when thoracic or epigastric injuries are present and should be treated according to existing recommendations [15].

The primary treatment goal for patients presenting with profound hypovolemic shock due to NCH after penetrating injuries is to maintain adequate cerebral and coronary perfusion and to prevent deterioration of hypovolemic shock into cardiac arrest before definitive haemorrhage control has been obtained. Both volume resuscitation and haemorrhage control are important to achieve this.

### Volume resuscitation

The evidence base guiding the indications and timing of pre-hospital volume replacement to maintain adequate coronary and cerebral perfusion in the pre-hospital setting is poor. Although a degree of hypotension is often accepted, and beneficial effects on outcome have been reported for permissive hypotension in the past [48], the effect may be due to both crystalloid restriction and haemostatic effects of lower blood pressures. The theory behind “permissive hypotension” is that it allows clot formation and prevents clot dislodgement by reducing the blood pressure gradient at the site of the injury and by mitigating the creation of a dilutional coagulopathy [49]. This may be more effective in patients with small-vessel bleeding rather than those with major arterial bleeding from penetrating injury, where the presumed pro-coagulant effects are likely offset by the reduction in stroke volume, blood pressure and hence coronary perfusion that is created. The ultimate endpoint of volume resuscitation is adequate coronary perfusion. However, there is no easy way to measure this, and various pragmatic surrogate end-points are often used in conjunction: consciousness, palpable pulses, ETCO2, DBP > 40 mmHg and absence of ECG signs indicative of myocardial hypoxia, such as ST segment changes and/or (broad complex) bradycardia.

When volume replacement is initiated, this should ideally be done utilizing a 1:1 ratio of PRBC’s and FFP’s (or freeze-dried plasma) to limit coagulopathy [50–52]. Whole blood has also been used and may have logistical advantages over component therapy in the pre-hospital setting [53]. Although the recently published RePHILL trial could not demonstrate a survival benefit for patients with haemorragic shock who received pre-hospital blood component therapy compared to those who received normal saline, the results should not be extrapolated to patients exsanguinating from NCH due to penetrating injuries, as these patients were significantly underrepresented in this trial [54].

The response to volume resuscitation should be monitored closely. Patients with haemorrhagic shock often demonstrate large variation in non-invasive blood pressure readings and automated readings tend to overestimate pressures [55]. Additional clinical indicators of perfusion (such as consciousness or palpable pulses) may be useful additional indicators of the requirement for pre-hospital transfusion [56]. Obtaining 4F femoral arterial access may be considered, especially when a longer journey to hospital is anticipated. This enables more reliable (invasive) blood pressure monitoring, and it also provides the ability to upsize to an 8-Fr sheath and proceed to REBOA should the patient deteriorate despite volume resuscitation. However, it may prolong scene time, which result in iatrogenic injury. Arterial cannulation is best done before leaving scene. Upsizing to a larger caliber sheath and even REBOA can be performed on route to hospital if needed.

### Haemorrhage control

Injured patients in extremis exsanguinating from NCH due to penetrating injuries may benefit from pre-hospital REBOA in addition to volume resuscitation when their injuries are situated below the level of the diaphragm to prevent them from dying before they reach definitive care [14]. However, to discern these patients is a challenge: no clinical factor in isolation can reliably define or predict exsanguinating haemorrhage. However, clinicians should have a high degree of suspicion when the mechanism of injury (MOI) is compatible with major vascular disruption, especially when shock is present immediately after the injury, and a constellation of clinical signs such as paleness, diaphoresis, air-hunger, venous collapse, hypotension (low volume or absent peripheral pulses), a low/falling ETCO_2_, tachy- or bradycardia and altered level of consciousness are present [57]. There are a number of validated algorithms that can be used to identify the need for large volume resuscitation [57–59]. However, few are incorporated into useful decision support systems that can be implemented in practice, and clinicians are often reluctant to trust such tools to support treatment decisions [60].

**Fig. 3:**
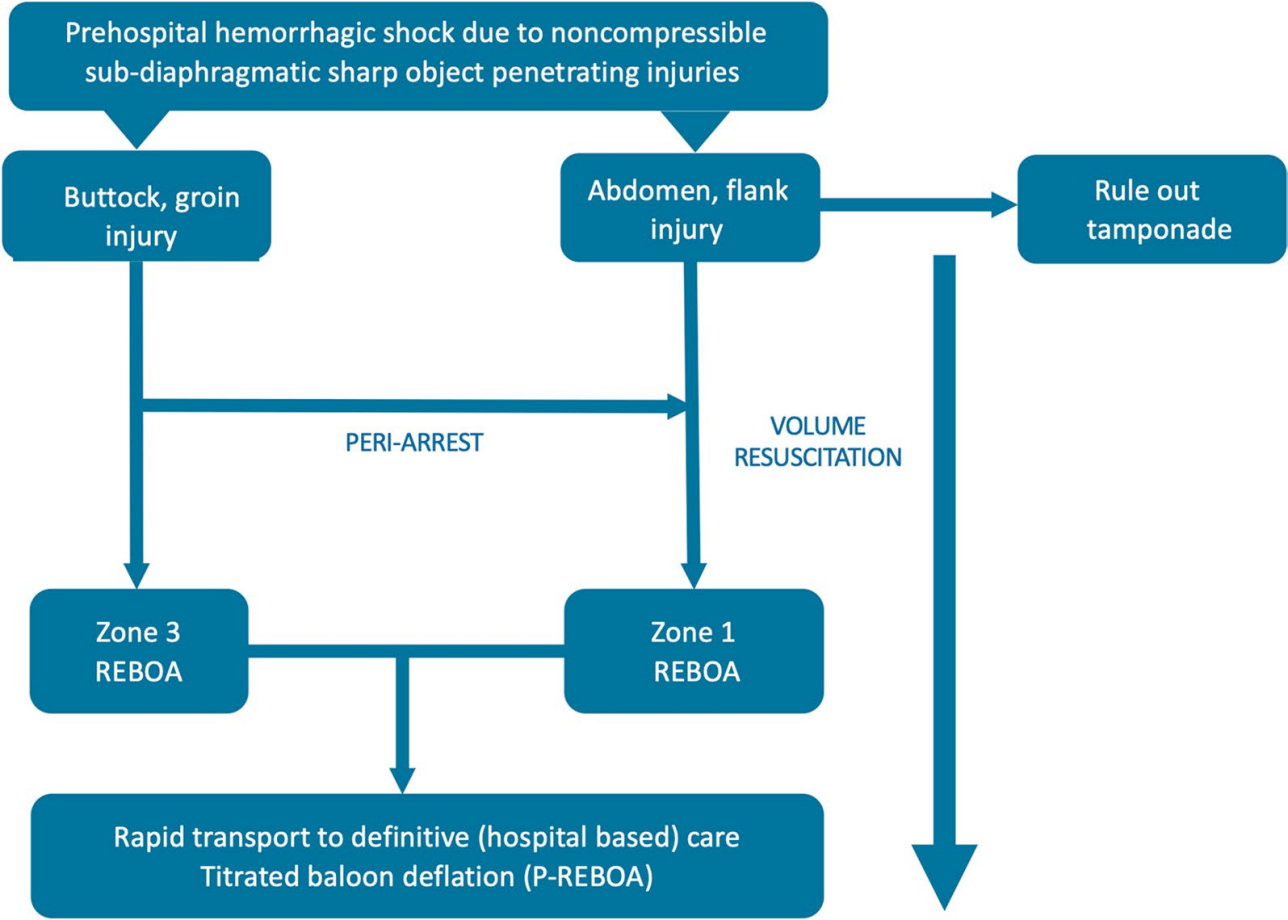
REBOA options for pre-hospital haemorrhage control and cardiac resuscitation for patients in haemorrhagic shock due to non-compressible penetrating injuries

Determination of the appropriate zone for REBOA (I or III) is based on anatomical location of the injuries and presenting physiology: penetrating injuries in the upper thigh, groin, or buttock are amenable to zone III REBOA, whereas for abdominal and flank penetrating injuries, the balloon is deployed in zone I [61]. In these patients, the presence of a (concomitant) pericardial tamponade should also be ruled out. When patients are about to arrest (bradycardia, falling ETCO2) despite all resuscitative efforts, zone I REBOA provides a higher afterload augmentation than zone III and may therefore be preferable [35] (Fig. 3).

Once the balloon is inflated, rapid transfer to a hospital with full trauma resuscitation capabilities is a priority. Titrated deflation (P-REBOA) and/or relocation of the balloon from zone I to zone III should be considered on route to hospital if injury location and physiology allow. If REBOA is not feasible in an exsanguinating patient, because of inability to cannulate the femoral vessels, pre-hospital RT with aortic occlusion can be considered in patients who are about to arrest.

### Emergency anesthesia and positive pressure ventilation

Pre-hospital emergency anaesthesia (PHEA) of hypotensive patients with penetrating injuries carries a significant risk, as it prolongs scene time, and previous studies have shown that PHEA in hypotensive trauma patients is associated with an increased in-hospital mortality [62]. This is likely related to the effect of induction agents on (an already compromised) preload, aggravated by subsequent positive pressure ventilation [63]. Therefore, where possible, PHEA should be delayed until immediate definitive haemorrhage control is available. If PHEA has to be carried out in the pre-hospital setting (actual or impeding airway obstruction, ventilatory failure), haemodynamically stable induction agents with a short half-life (t_1/2_K_eo_) such as ketamine are preferable [64]. Sometimes successful REBOA in patient in (peri)arrest may increase cerebral perfusion and the level of consciousness and make sedation and analgesia necessary.

## Knowledge gaps and future practice

Services that have adopted advanced techniques are rapidly developing experience on which groups of patients may benefit from them and how techniques can be adapted to facilitate delivery. There are some key questions that remain unanswered.

### “Who needs interventions on scene”?

Historically, the penetrating trauma literature suggested that short scene time and fast transport to hospital defined effective pre-hospital management [65], and pre-hospital time has been associated to in-hospital mortality [66, 67]. Recently, the Eastern Association for the Surgery of Trauma (EAST) reported an increased odds of mortality for patients who underwent one or more pre-hospital procedures [68]. However, it is important to note that these studies were based largely on gunshot wounds and did not take into account the availability of advanced pre-hospital therapeutic options to minimize or replace blood loss, such as REBOA, RT and pre-hospital transfusion of blood products. Although it is important to minimize scene time and prevent interventions associated with an adverse outcome (such as pre-hospital PHEA) [62], a small, critically ill subset of patients require advanced interventions on scene to survive until hospital [14, 69]. Based on available literature and our experience, we can recommend RT for suspected cardiac tamponade following penetrating trauma with < 10 min of TCA. Both RT and REBOA are likely futile in patients with an asystolic arrest from exsanguination. Both procedures can be considered in exsanguinating TCA (to prevent asystolic arrest), and REBOA can be considered in patients with a profound hypovolemic shock to prevent TCA. Identifying the latter group of patients is challenging as validated clinical criteria to predict imminent exsanguination (and thereby the need for advanced pre-hospital interventions) do not exist. Future research should focus on how these patients can best be identified at the earliest opportunity (for example with artificial intelligence assisted algorithms [70]), so that appropriate treatments can be efficiently implemented without significant delay in transport to hospital, while at the same time we don’t cause harm by performing RT or REBOA on patients unlikely to benefit from these interventions, and we don’t delay transport to hospital in (the majority) of patients who don’t need these advanced pre-hospital interventions.

### “How should we treat TCA after subdiaphragmatic injuries”?

At present, experience with REBOA for patients in TCA with subdiaphragmatic injuries is limited. If advanced pre-hospital interventions at all are provided to these patients, the most likely interventions is a RT. Pre-hospital survival rates of this specific cohort are unavailable, but from in-hospital studies we know that reported survival rates of this patient group of RT patients are extremely poor [71]. REBOA is less invasive and has the additional advantage over RT that P-REBOA can be initiated after the initial resuscitation phase. Various case series have demonstrated the feasibility of using REBOA for non-traumatic cardiac arrest in the pre-hospital setting [36, 37], and a prospective trial (REBOARREST [72]) is presently being conducted in Scandinavia to investigate this further. At the same time, the P-PRO study, a feasibility study of zone I P-REBOA ^47^, is investigating this in patients with exsanguinating sub-diaphragmatic haemorrhage and imminent risk of hypovolemic cardiac arrest, or recent hypovolemic cardiac arrest. The results of these studies will hopefully provide some direction on the future treatment of patients in full cardiac arrest after subdiaphragmatic injuries.

### “What is the best mode of transport to definitive care”?

When the decision has been made to convey the patient to hospital, the most suitable available transport modality should be chosen. In urban areas with short distances to trauma centers, this will usually be a ground ambulance. For patients attended in rural areas, the decision may be more complex and may depend on many factors, such as available resources, weather, injuries identified and anticipated clinical course. Although helicopter transport may contribute to a shorter time to definitive care and has been shown to be associated with an improved survival after major trauma in general [73], a survival benefit compared to ground transport has not been shown for this specific patient category [74]. Further, cabin space in some aircraft types does not allow advanced procedures to be carried out in flight, mandating careful consideration of all potential clinical scenario’s before committing to air transport.

### Limitations

It is important to note that the pre-hospital response (both for patients in full cardiac arrest and for patients in profound shock) should always be adapted based on the expertise available. This covers seniority, training and the number of clinicians in a team required to deliver interventions in a timely fashion. Advanced interventions as described in this paper can only be provided in a system with ample opportunities to train and maintain skills. They mandate a system of rigorous clinical government, to deploy these techniques safely, especially as the evidence base behind many of the techniques described is still small. Even in the hands of well-trained providers, these interventions have the potential to delay scene times and may come with significant (potentially life threatening) complications [16, 17]. Therefor a dynamic assessment of potential risks and benefits is of utmost importance before these procedures are carried out in the pre-hospital setting.

## Conclusion

Pre-hospital resuscitation of patients in extremis due to NCH from non-ballistic injuries is dependent on available resources. Although minimizing scene time is important, several advanced pre-hospital interventions can be provided to a select group of exsanguinating patients in the pre-hospital setting in order to improve their chances of survival. Research studies as well as the experience gained from the adoption of these advanced resuscitation interventions by some pre-hospital services provide guidance on the future resuscitation of this patient group.

## Data Availability

Not applicable (as this is a review, and no original data were generated).

## Abbreviations

ERC: European Resuscitation Council
FFP: Fresh frozen plasma
HEMS: Helicopter emergency medical services
POCUS: Point of care ultrasonography
PRBC: Packed red blood cells
REBOA: Resuscitative endovascular balloon occlusion of the aorta
ROSC: Return of spontaneous circulation
RT: Resuscitative thoracotomy
